# Characteristics and patients’ portrayals of Norwegian social media memes. A mixed methods analysis

**DOI:** 10.3389/fmed.2023.1069945

**Published:** 2023-03-16

**Authors:** Anders Hagen Jarmund, Sofie Eline Tollefsen, Mariell Ryssdal, Audun Bakke Jensen, Baard Cristoffer Sakshaug, Eirik Unneland, Berge Solberg, Bente Prytz Mjølstad

**Affiliations:** ^1^Department of Clinical and Molecular Medicine, Norwegian University of Science and Technology (NTNU), Trondheim, Norway; ^2^Department of Public Health and Nursing, Norwegian University of Science and Technology (NTNU), Trondheim, Norway

**Keywords:** social media, professional identity development, professionalism, patient, health care education, e-professionalism

## Abstract

**Background:**

Despite reports on troublesome contents created and shared online by healthcare professionals, a systematic inquiry of this potential problem has been missing. Our objective was to characterize the content of healthcare-associated social media memes in terms of common themes and how patients were portrayed.

**Materials and methods:**

This study applied a mixed methods approach to characterize the contents of Instagram memes from popular medicine- or nursing-associated accounts in Norway. In total, 2,269 posts from 18 Instagram accounts were included and coded for thematic contents. In addition, we conducted a comprehensive thematic analysis of 30 selected posts directly related to patients.

**Results:**

A fifth of all posts (21%) were related to patients, including 139 posts (6%) related to vulnerable patients. Work was, however, the most common theme overall (59%). Nursing-associated accounts posted more patient-related contents than medicine-associated accounts (*p* < 0.01), but the difference may be partly explained by the former focusing on work life rather than student life. Patient-related posts often thematized (1) trust and breach of trust, (2) difficulties and discomfort at work, and (3) comical aspects of everyday life as a healthcare professional.

**Discussion:**

We found that a considerable number of Instagram posts from healthcare-associated accounts included patients and that these posts were diverse in terms of contents and offensiveness. Awareness that professional values also apply online is important for both healthcare students and healthcare providers. Social media memes can act as an educational resource to facilitate discussions about (e-)professionalism, the challenges and coping of everyday life, and ethical conflicts arising in healthcare settings.

## 1. Introduction

The arrival and spread of online social media have introduced possibilities and challenges for society all around the world. For healthcare students and professionals, the implications of online presence and behavior are still emerging and e-professionalism is a construct comprising “the attitudes and behaviors (some of which may occur in private settings) reflecting traditional professionalism paradigms that are manifested through digital media” (1). Unfortunately, studies have revealed that e-professionalism is difficult, especially for students (2–6), and concerns have recently been raised across countries that certain forms of online humor published by healthcare workers conflict with professional values (7–10). These concerns have, however, been anecdotal in nature and systematic characterization of such material is lacking.

Humor is a complicated matter in terms of professionalism and serves multiple functions for healthcare professionals. It can facilitate communication, support therapeutic processes or act as a strategy to cope with demanding situations and difficult emotions (11, 12). By sharing challenging experiences through jokes, healthcare workers remind each other that struggling and making mistakes are common, without afflicting shame or guilt (7). However, not every form of humor aligns with the professional norms in healthcare. Stigmatized groups seem to be especially vulnerable to ridicule (13). Dark humor, ridiculing tragic events and suffering can be a useful tool in the face of distress, but may appear uncanny, hostile or offensive from the outside (14). In some cases, humor can become abusive or degrading in respect of vulnerable patients (15, 16). Thus, there has been a call for the education of healthcare professionals to also address the use of humor (17) as part of the wider “hidden curriculum” (18).

Memes constitute a genre of humor that has gained attention in relation to troublesome online contents (7–9). A meme is typically an image or short video annotated with text shared in social media. Examples are not reproduced here for legal reasons, but illustrative examples have been published by Berre and Peveri (9), Harvey (7), and Song and Crowder (10). The social media platform Instagram, which is intended for image and video sharing, has about 2.8 million users in Norway, corresponding to 67% of the adult population, and more than half of those between 18 and 50 years of age report daily use (19). The use is highest among young women (18–29 years) where 89% has an Instagram account. The potential for wide outreach is thus considerable and problematic contents produced by healthcare students have already caused concerns among educators (9). The lack of systematic knowledge regarding the contents of these images and videos makes it impossible to assess the prevalence of problematic material and restricts how educators can thematize this phenomenon in terms of e-professionalism.

To address the need for systematic descriptions of social media memes, this paper employs a mixed methods approach to characterize Norwegian healthcare-associated memes posted on Instagram. The aim of this study is to provide systematic knowledge to guide and support public discussions regarding healthcare professionalism and humor in social media, and to identify areas where social media memes can be used as a resource for professional identity formation in healthcare education.

## 2. Materials and methods

### 2.1. Data collection

Google was used to search for an initial list of relevant accounts (search queries: “medisin memes site:instagram.com” and “sykepleie memes site:instagram.com”). The search was conducted on June 16th, 2021. For each account with less than 500 followers, the lists of followers and followings were manually reviewed, and relevant accounts noted. The process was repeated until no more relevant accounts could be identified. Accounts were included in the study according to the inclusion and exclusion criteria in Table 1 and categorized as related to nursing or medicine, and the number of followers and followings was recorded. From the selected accounts, all posts published prior to June 1st, 2021 were assessed for eligibility. The delay between June 1st and 16th was assumed enough for the posts to receive representative reactions in form of likes and comments. Images, videos, date, caption, and the number of likes and comments were extracted for each post. The publication date of the first post from each account was used to calculate account age.

**TABLE 1:** Inclusion and exclusion criteria for relevant Instagram accounts.

Inclusion criteria	Exclusion criteria
Name or description refers to medicine or nursing	Mentions specific persons in name or description
Primarily publishing memes	
Public	
Has more than 100 followers	

The study was approved by the Norwegian centre for research data (NSD, reference number 128255) and the included accounts were notified and received written information about the study in line with privacy regulations.

### 2.2. Quantitative analysis

The quantitative analysis aimed to (1) characterize the popularity of various themes and (2) explore whether specific themes affect the response to the posts. Codes were developed by two authors in collaboration from a set of 100 randomly selected posts and independently validated by three coders. The inter-rater reliability of each code was assessed by Gwet’s Agreement Coefficient 1 (AC_1_). AC_1_ is robust to the Kappa Paradox where Cohen’s Kappa underestimates agreement in the case of skewed data, i.e., when the prevalence of some codes is small (20). The codes were refined and independently tested until satisfactory inter-rater reliability was reached (AC_1_ > 0.40), except for codes that were expected to show large inter-rater variability (i.e., *Vulnerable patient* and *Offensive*). Next, each post was randomly assigned to three independent coders. The coders had an option to flag posts for review if they were difficult to code and posts were excluded if all three coders found it difficult to assign suiting themes. To improve validity, only codes applied by ≥ 2 coders were kept for analysis. All posts marked for review were evaluated by two authors in collaboration and recoded.

The proportion of posts belonging to each theme was calculated by account to compensate for the varying number of published posts. These proportions were used to calculate correlation between themes (Spearman’s correlation coefficient) and compare prevalence between professions (Kruskal–Wallis test).

Linear mixed models were used to assess the effect of specific themes on the number of reactions (likes and comments). The number of reactions to a post depended on the number of followers of the account at the time of posting. To account for this, we devised a case-control comparison by selecting four control posts for each theme-related post (case). For each post related to a specific theme (e.g., student life), the two previous and next posts *not* related to that theme were selected from the same account (Supplementary Figure 1). Thus, multiple case-control groups of 3–5 posts were created for each theme. Next, the number of reactions was standardized by dividing on the standard deviation of the corresponding control posts. The regression coefficient can then be interpreted in terms of how many standard deviations a specific theme will increase or decrease the number of reactions. Nested clustering (case-control group nested within account) was included in the model as a random intercept. Profession and theme were included as fixed effects, as well as their interaction. Independent models were fitted for the number of likes and comments.

Bootstrapping was used to estimate confidence intervals for the regression coefficients. The case-control groups were stratified by account and resampled with replacement. Next, new linear mixed model regression coefficients were estimated from the bootstrapped samples. Finally, the 2.5 and 97.5% percentiles were extracted and regarded as 95% confidence intervals for the coefficients.

All calculations were conducted in R version 4.0.2 (21) and *p*-values were adjusted within test with the Benjamini-Hochberg procedure. Visualizations were made with the UpSetR (22), corrplot, and ggplot2 (23) packages for R.

### 2.3. Qualitative analysis

Qualitative analysis aimed to provide rich descriptions of how the memes portrayed patients and their relatives and to explore characteristics of professionally problematic posts. To this end, 15 problematic posts and 15 unproblematic posts were systematically selected for focused discussions.

To identify problematic and unproblematic posts, the posts (*n* = 491) including patients/relatives were scored by offensiveness on a numerical rating scale from 0 (not offensive) to 10 (highly offensive) by at least three authors. The 15 posts with highest and lowest mean score were considered most and least offensive, respectively, and selected for comprehensive qualitative analysis.

The qualitative analysis was conducted using a methodology originally designed for analysis of press photograph story (24) and later adapted for social media analysis (25). Four authors jointly reviewed all selected posts through focused discussions and the following features were detailed for each selected post (Supplementary Table 2): (1) Uninterpreted content, (2) Text, (3) Interpreted content, (4) Humor, (5) Caption, (6) Offensiveness, and (7) Theme. Two experienced qualitative researchers (BPM and BS) reviewed the selected posts independently to cross-check that identified themes corresponded to the overall impression. A consensus on the most prominent message in each meme was achieved through thorough discussion.

## 3. Results

### 3.1. Accounts and posts

After the initial Google search and review of lists of followers and followings, 51 accounts were assessed for eligibility. Of them, 18 accounts were included and categorized as related to medicine (*n* = 5) or nursing (*n* = 13). Account characteristics are shown in Table 2. In total, 2,319 posts had been published prior to June 1st, 2021. The median (range) number of posts per account was 96 (6–596). Not all accounts were actively publishing posts at the time of the study. The median (range) time span from the first to the latest post was 284 (14–979) days, and the median (range) number of posts per month was 11.9 (2.2–86.8).

**TABLE 2:** Account characteristics by profession.

	Nursing	Medicine
Accounts, sum, *n*	13	5
Followers, median (range), *n*	1,131 (254–44,631)	1,161 (954–2,987)
Following, median (range), *n*	69 (10–1,811)	121 (43–225)
Age, days	172 (14–979)	284 (205–637)
Published posts, sum, *n*	1,879	440
Included posts, sum (% of published), *n*	1,835 (98%)	434 (99%)

In total, 16 posts were marked for review by all three coders and excluded from further analysis, whereas 227 were flagged for review by one or two coders and recoded by two authors in collaboration. Thirty-four posts were excluded during recoding, leaving 2,269 posts for further analysis. Of these, 14 posts did not reach majority on any codes but have been kept in Table 2 as they were not flagged for review during coding. A flow chart of post inclusions and exclusion can be found in Supplementary Figure 2.

### 3.2. Quantitative analysis

Eleven general themes were identified and are described in Table 3. The posts were coded by three authors, resulting in high inter-rater reliability (AC_1_ ranging 0.77–1.00, adjusted *p* < 0.001, Supplementary Table 1).

**TABLE 3:** General themes identified in the memes and their effect on the number of likes (B_*likes*_) and comments (B_*comments*_).

Theme	Example	B_*likes*_ [CI]	B_*comments*_ [CI]
Advertisement	A specific product is mentioned as the solution when the electronics are failing, with a lifeguard running to “save the day”	–0.57 [–1.45, 0.26]	14.36 [–0.01, 31.71]
Academic concept	Two children captioned Bax and Bcl–2 (proteins involved in apoptosis) are playing with Bax hitting Bcl–2 in the head with a bottle captioned “apoptosis”	**–0.44** **[–0.82, –0.12]**	–0.12 [–0.40, 0.15]
Corona	A man running with the caption indicating that there is focus on COVID–19 and that someone has coughed	–0.16 [–0.43, 0.10]	–0.13 [–0.41, 0.20]
Exams/Tests	A child gradually disappearing with the caption indicating that a subject is not relevant for the exams	–0.17 [–0.55, 0.22]	**1.18** **[0.39, 2.10]**
In-jokes	Picture of a possible lecturer with a celebrity in a panel next by	**0.59** **[0.24, 0.95]**	**1.85** **[0.20, 3.61]**
Internship	Mr. Bean proudly displaying a card, with the caption indicating that a student has received his first hospital identification card	**0.32** **[0.07, 0.58]**	**0.81** **[0.22, 1.38]**
Offensive	A man offering olanzapine (antipsychotic medication) to the sad relatives of a patient	0.05 [–0.52, 0.61]	0.40 [–0.19, 1.09]
Patient	An older person is told he has a wife and a family, to which he responds “Well shit”	**0.38** **[0.19, 0.56]**	**0.37** **[0.15, 0.60]**
Private life	A man pointing with the caption indicating he is pointing out medical errors in television shows	0.12 [–0.14, 0.40]	**0.44** **[0.09, 0.81]**
Student life	A crying woman with the caption indicating that she is trying to catch up with the curriculum	**0.41** **[0.07, 0.75]**	0.36 [–0.01, 0.78]
Vulnerable patient	A nude man hanging from the roof, with a caption indicating that he is a patient who is refusing hospitalization despite his apparent need for it	**0.59** **[0.19, 0.94]**	**0.98** **[0.27, 1.76]**
Work	Leaving a chaotic scene with the caption indicating shift change	0.07 [–0.10, 0.36]	**0.90** **[0.67, 1.12]**

B_*likes*_ and B_*comment*_ refer to standardized regression coefficients estimated from linear mixed models (see text for details) and positive coefficients reflect the theme being associated with an increase in the number of reactions. Confidence intervals (95% CI) are estimated from bootstrapping and robust effects (95% CI not containing zero) are indicated in bold.

**FIGURE 1:**
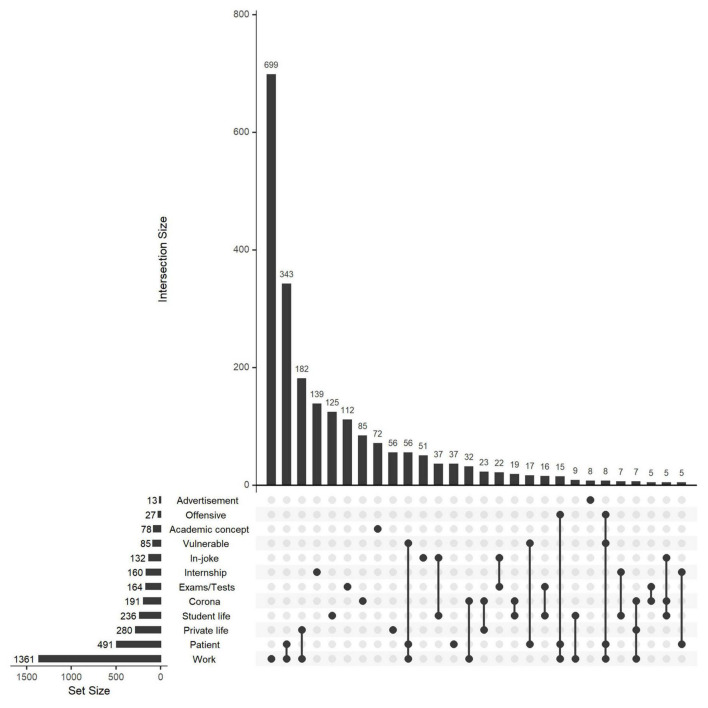
Number of posts related to common themes. The total number of posts related to each theme is shown to the left, whereas the upper bar plot shows the intersections between various themes (e.g., 343 posts were related to both work and patient). Only intersections with ≥5 posts are shown.

**FIGURE 2:**
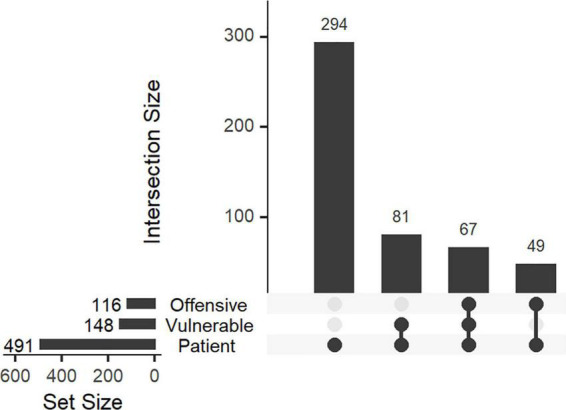
Number of patient-related posts coded as vulnerable or offensive. The total number of posts related to each theme is shown to the left, whereas the upper bar plot shows the intersections between various themes (e.g., 67 posts were relating to vulnerable patients and considered offensive).

The occurrence of various themes is illustrated in Figure 1 for all accounts and in Supplementary Figure 3 for medicine- and nursing-associated accounts separately. Most posts were related to work, either alone (*n* = 699) or in combination with patients (*n* = 422) or private life (*n* = 183). In total, 491 posts were patient-related (Figure 2). Of these, 116 posts were regarded as offensive (24%), 148 posts (30%) as depicting vulnerable patients, and 67 posts (14%) as an offensive depiction of a vulnerable patient. There were significant correlations between some themes (Supplementary Figure 4): Accounts posting about work tended to post about vulnerable patients and patients in general, but not in-jokes or about student life or exams. Accounts posting mostly about student life, on the other hand, tended to post in-jokes and about exams, but less about patients or work.

**FIGURE 3:**
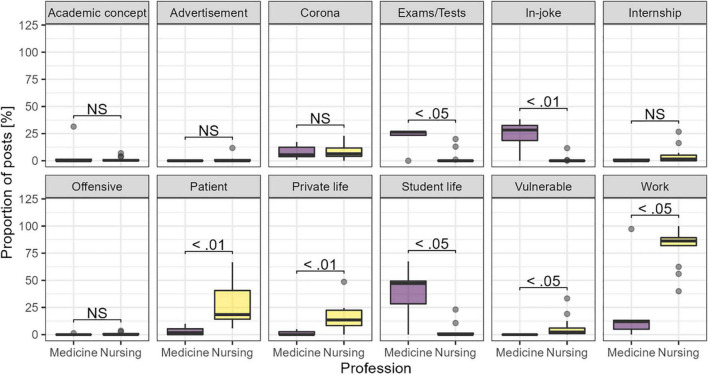
Proportion of posts related to each theme, by the assumed professional belonging of the accounts. Adjusted *p*-values from the Kruskal–Wallis test.

Accounts related to medicine or nursing showed significant differences in the number of posts related to several themes. The relative occurrence of various themes is shown in Figure 3. Posts from medicine-associated accounts were more often about exams (*p* < 0.05), student life (*p* < 0.05) or in-jokes (*p* < 0.01). In contrast, posts from nursing-associated accounts were more frequently related to work (*p* < 0.05), private life (*p* < 0.01) or patients, both vulnerable (*p* < 0.05) and in general (*p* < 0.01).

**FIGURE 4:**
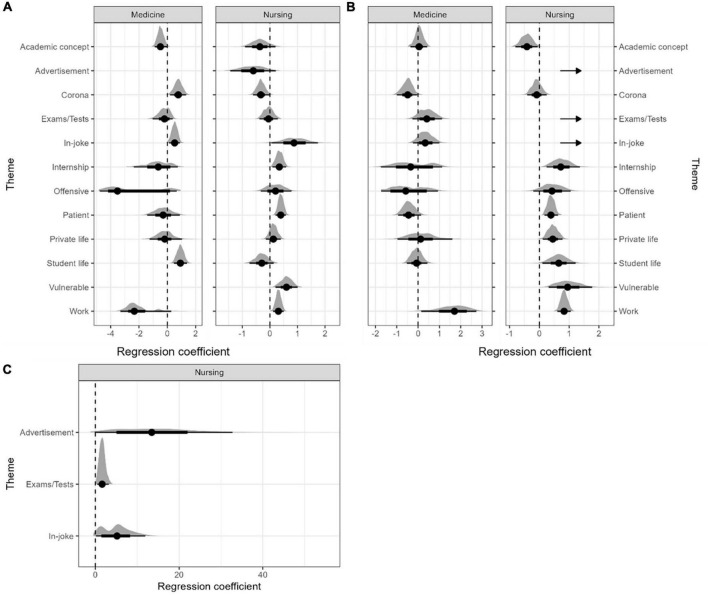
Effect of theme on the number of **(A)** likes and **(B,C)** comments. The regression coefficients are from linear mixed models and represent the deviation from the mean in terms of standard deviations of nearby posts without the specified theme. The distribution shows the robustness of the estimates as calculated by bootstrap validation. The dotted lines indicate no effect. Some themes were separated **(C)** to avoid skewing of scale, as indicated by arrows in panel **(B)**. For medicine-associated accounts, the number of posts related to advertisements (4 posts) and vulnerable patients (1 post) were too low to yield interpretable estimates.

Overall, theme had only minor effect on the number of reactions as shown by the regression coefficients given in Table 3 and shown by profession in Figure 4, with some exceptions. The strongest effect was seen for advertisements that had a clear tendency to have more comments than other posts. However, due to a low number of such posts, the effect was not robust to bootstrapping and was only estimable for nursing-associated accounts (Figure 4). Posts containing in-jokes or relating to exams/tests also tended to have more comments, but the effect was much weaker than for advertisements (Table 3). Although the effect of theme on number of likes was overall weak (<1 standard deviation compared to control posts), posts with in-jokes or relating to vulnerable patients tended to have more likes than other posts. In contrast, posts depicting academic concepts tended to receive fewer likes. When assessed by profession, a similar pattern emerged (Figure 4). In medicine-associated accounts, posts about work or coded as offensive tended to receive fewer likes. In contrast, posts about work received more comments in both medicine- and nursing associated accounts and some more likes in nursing-related accounts. There was a tendency for patient-related posts to receive more likes and comments in nursing-associated accounts, whereas this effect was absent for medicine-associated accounts.

### 3.3. Qualitative analysis

#### 3.3.1. The depiction of patients: How and who

The 30 selected posts showed a rich variation in graphical techniques and the use of symbols. The largest portion of posts contained cartoons or snapshots, either pictures or videoclips, from popular culture (e.g., scenes from TV series) with added explanatory text and captions. Few posts depicted actual situations involving healthcare. Instead, patients and healthcare workers were typically represented by other characters, using text captions to convey the setting and the roles. Both patients and healthcare workers were sometimes depicted as animals. There were, however, examples of what may have been authentic patients, in ambulance or in hospital, and a photo taken inside a Norwegian healthcare institution (the photo did not show any patients or sensitive information). Some posts made use of more advanced symbolism, such as the trojan horse.

Some groups of patients were repeatedly depicted in the 30 selected posts. These were typically vulnerable patients such as confused or fragile elderly patients, patients suffering from psychosis or delirium, and drug-affected or agitated patients. The healthcare worker was often anonymous, and profession and position were typically not stated explicitly.

The point of view varied between posts. Often, the character representing a healthcare worker was marked with personal pronouns such as “me”, “I”, or “you”. In others, we observed the situation as an unnamed third party. Another common configuration was a photo or video representing the patient’s response to an action, captioned “every time you [do something to the patient]”. The patient was referred to as “me” in only one of the 30 selected posts.

#### 3.3.2. Thematic analysis: Main themes

Three overarching and recurring themes emerged during the analysis of posts considered the most or least offensive. Below we present main themes and related subthemes from the thematic analysis with illustrative examples demonstrating how the themes manifest themselves in distinct ways in posts considered offensive when compared to posts considered innocent.

##### 3.3.2.1. Trust and the breach thereof

Many posts involved some form of breach of trust. This was thematized in various and diverse ways, often in the shape of deception: healthcare workers lying, omitting, pretending. Among the most offensive posts, this theme was frequently connected to administrating medications, typically antipsychotics or sedatives. An illustrative example: *a healthcare worker saying “I am just flushing your venous catheter” whereas the syringes are clearly marked with antipsychotics*. In one such post, the healthcare worker additionally calms the patient by, falsely, saying *“Yes, it is only salt water”*. Another form of pretending was demonstrated by a slow code scenario where an elderly patient receives incomplete and superficial chest compression from a healthcare worker while the relatives are crying in the background. Some of the more innocent posts also touched upon forms of deception, such as concealing feelings in front of the patient or pretending to be working while hiding from tiresome patients or relatives.

Dealing with unprofessional thoughts, fantasies and feelings related to patients was considered a distinct aspect of managing trust as a healthcare worker. This spanned from expressed desire to hurt and punish patients for being difficult and enjoying that they struggle to frustration over patients, not prioritizing what is best for the patient, and looking at patients’ bodies with un-caring eyes.

##### 3.3.2.2. Difficulties and discomfort at work

Almost all the discussed posts depicted situations at work that involved some form of difficulty or discomfort. In contrast to posts considered offensive, innocent posts typically revolved around challenges encountered at work as a healthcare professional and with patients having passive roles such as observers or extras or were just referred to. Examples include *doing heavy lifting alone, hiding from and avoiding patients, feeling incompetent or as an imposter*, and *struggling with a task in front of a patient*. One post stood out as more confession-like than a meme: *a healthcare worker described being sexually assaulted by a patient and, subsequently, laughed at by colleagues when searching support*. In posts considered offensive, on the other hand, the patients were often portrayed as the direct cause to the discomfort or challenge. A post considered offensive depicted *a healthcare worker entering a patient’s room where the patient is lying exhausted on the floor with hands covered by feces, which have also been smeared onto the walls. A text caption informs that the patient had previously refused to receive assistance.*

Many posts thematized how difficulties at work were solved in less-than-optimal ways, often involving breach of trust as described above. Uncooperative patients and patients using long time to perform basic tasks – delaying or creating “difficulties” for the healthcare worker – tended to be met with frustration, anger, force, and deceit.

##### 3.3.2.3. The comedy of everyday life as healthcare professionals

Another distinct theme emerged from work-situated posts that did not involve discomfort or difficulties but rather focusing on absurdity or surprise. A subgroup of the posts that were considered innocent which depicted small, everyday incidents such as *a patient being wakened by the alarm of an infusion pump, a healthcare worker telling the same joke to multiple patients, or a healthcare worker accidently making noises when checking up on a sleeping patient*. These posts often implied deep compassion for the patient or an unspoken alliance between patient and healthcare worker. For example, several posts showed *the administration of medicine where the dosage is far too low to sufficiently help the patient*. This was, however, framed as the fault of an absent doctor, leaving both the depicted healthcare worker and patient in shared helplessness.

In the offensive group there were posts where the comedy was entirely on the patient’s behalf, such as *psychotic patients doing or saying allegedly strange, ridiculous, or stupid things or patient’s angry responses to naloxone* (an antidote to opioids). These posts were considered more malign. An interesting contrast was the depiction of *an elderly patient happily and eagerly folding hospital towels*. Despite this being humor on the patient’s behalf, it was perceived as more compassionate than ridicule and was part of the group of posts considered innocent.

#### 3.3.3. Humor based on whose pain?

Systematic differences emerged between posts considered as offensive or not, regarding whose expense the post’s humor was based. In many of the offensive posts the patients were subject to an action by a healthcare worker. Consequently, the humor was at the expense of the patient and the patients’ vulnerability was an important part of the humorous element of the post. This is exemplified by the repeated theme of deceitful administration of medication to patients, often depicted as either psychotic or demented. In the innocent posts, on the other hand, the patients were not negatively affected by the actions of the healthcare worker, and the patients were mostly supporting characters in the situations depicted. Here, the “pain” was clearly at the expense of the healthcare worker. However, the focused discussions revealed that these differences were not always obvious. For example, some of the posts considered to be offensive and involving pain on the patient’s expense could be interpreted as displays of the power- and helplessness healthcare workers may experience when facing specific patients.

## 4. Discussion

Despite growing concerns regarding e-professionalism among healthcare students and professionals, the contents of social media humor from these groups have evaded systematic characterization. To fill this gap, we employed a mixed methods approach to map important themes both quantitatively and qualitatively. The examined memes showed diverse, yet characteristic, forms of humorous contents and clear differences were found between professions. While nursing-associated accounts had large audiences and focused on themes related to work-life, the medicine-associated accounts had smaller outreach and focused on student-life. Theme had only minor effects on the number of reactions and comments. The most offensive posts included vulnerable patients such as elderly patients and people with mental disorders or drug-addictions, whereas the least offensive posts thematized challenges as a health-care professional and the comedy of everyday life. Although the patient-related content comprised only a minor subset of the material, many problematic examples were found, and those regarded as most offensive were found to jeopardize the trust between patients and healthcare professionals. It should, however, be noted that none of the included posts broke the duty of patient confidentiality or were found so problematic that further steps were considered.

The accounts belonging to the different professions (medicine and nursing) were clearly targeting distinct audiences: the nursing-associated accounts targeted mainly nurses in working positions whereas the medicine-associated accounts targeted student populations. This notion is supported by the medicine-associated account names often referring to universities. It is possible that the shorter duration of the nursing education, with frequent separation into internships at various places, leaves less room for a meme culture to form. The relatively small subset of student-targeted nursing accounts have, however, caused ethical concerns (9). Another possible explanation is that the number of working nurses (about 50,000, excluding midwives and specialist nurses (26)) is larger than the number of nursing student (about 5,000 students (27)). The relative lack of medicine-associated memes from working physicians may reflect professional maturation during the study or that other platforms or private accounts are used. Shedding light on the “hidden curriculum” has been recognized as an important step to fully integrate professional identity formation as part of healthcare educations (18) and our findings suggest that refining e-professionalism cannot be a process isolated to educational institutions but must include professional bodies reaching healthcare practitioners as well.

The professional tension accompanying social media has manifested itself during the last decade, and along with it the discussion of how healthcare professionals should conduct themselves on such platforms, so-called e-professionalism. One extreme approach to this may be to conclude that all public online depictions of patients produced by healthcare professionals are dubious. Being or feeling seen, exposed, looked at, or deprecated by others are central components of shame (28, 29), and reminding the patient that one is constantly observed, evaluated, thought about, and discussed may induce self-consciousness and perhaps evoke both shame and a sense of betrayal or alienation – especially if one is negatively portrayed or the perspective conflicts with one’s own experiences. We found several examples of this, such as healthcare professionals experiencing discomfort when meeting or observing a patient or finding a patient laughable in appearance or behavior. Depriving patients the control over how they are imagined, portrayed, and spoken about may add to their powerlessness in face of a healthcare system where their social and bodily control has, often, already been weakened. Trust is one of the pillars of professionality (18) and healthcare professionals are obliged to guard patient integrity in all situations and this commitment conflicts with the creation of humorous memes. This view invites students of healthcare professions to reflect upon reasons to why collapses in (e-) professionalism may occur and why one might be tempted to expose or ridicule a patient. In addition, one of the expressed concerns relating to the social media memes has been the possible normalization of problematic attitudes among students. The memes can become memorable and influential parts of the so called “hidden curriculum” of healthcare education (7) which is now recognized as an integral part of how professionalism develops (18). The repeated exposure of vulnerable patient groups, such as patients suffering from dementia or psychiatric or addiction disorders, that was identified in the current study may contribute to an “othering process” similar to what have been seen during the COVID-19 pandemic (30). Another possible route of harm is that “these memes can distort our senses, blunting our abilities to detect human vulnerability and, in so doing, poison the relational ethics of our practice” (8). These concerns are, however, not unique to medical memes and pertain to all use of humor in healthcare settings (16).

A contrasting view may be that the production of humorous memes are important forms of self-expression that, if they manage to maintain patient confidentiality, are creative ways to identify, communicate, and cope with problems and challenges arising in professional life. Creative artmaking is an effective way to explore issues related to professional development and visual arts offer distinct benefits compared to verbal reflection (31). Patients are not to be infantilized but should be respectfully treated as ordinary people, which may include that unflattering behavior is commented and pointed out – not as an act of humiliation but to help refine patients’ ability to mentalize and know how they appear to others. Thus, the memes can possibly serve honorable causes, including as educational tool or as a way to cope or vent (7, 8, 10). The empowering and positive potential of healthcare-associated memes is illustrated by memes produced by or for patients [e.g., (32–37)]. This view invites students of healthcare professions to explore how humor and social media can be used in constructive ways to raise awareness about challenges encountered at work and as an alternative and casual way of communicating with (specific groups of) patients. The fact that most of the memes analyzed by us relate to work or student life – and often frustrating sides of these, such as work-spare time conflicts or exams – suggests that the memes are primarily a way to vent. Especially, the memes can be used as vehicle to communicate experiences that are not easily shared otherwise, such as shame (38), the embarrassment from making mistakes (7) or being disempowered (10). These are common yet painful and vulnerable experiences among healthcare workers that may be eased by establishing them as shared experiences that can be joked about. Thus, educators may seek to “help students and trainees to find an authentic voice, based at least in part on the profession’s ideals, that works in both medical and non-medical life-worlds” (39, 40) so that the memes can remain useful while adhering to professional standards. The Medical Education e-Professionalism (MEeP) framework is a research-based attempt to define core competencies for healthcare professionals in relation to digital space (41). Here, developing professionality involves recognizing the mission and social contract of the medical profession, and specific competencies are described along the axes of professional values, behaviors, and identity formation. The framework has been shown useful to guide implementation of e-professionalism education (42).

The qualitative analysis revealed that problematic posts often depict conflicts between normative and descriptive ways of providing healthcare services. Although all healthcare professionals are trained to know the importance of patient respect, confidentiality, and trust, one might find oneself in situations where the highest professional standards cannot be met due to organizational (e.g., high workload or understaffing) or personal (e.g., inexperience, anger, or frustration) reasons and where techniques such as deceit are found necessary. These illustrations may have educational value that can enlighten healthcare professionals and administrators about unpleasant pragmatism arising from how the services are organized. From a patient perspective, however, the unpleasant pragmatism may lower the public’s trust in the healthcare services. Nevertheless, healthcare professionals must reconcile human imperfections and organizational limitations with the demands of professionalism, and keeping patient-directed humor at spatial and temporal distance from patients – such as between colleagues in the lunch room – has being suggested as an acceptable but controversial solution (16, 43). With online social media, however, spatial and temporal distance collapses and the borders between private and public are blurred (1). All the Instagram accounts included in this study were public accounts, accessible for everyone. For some, deciding to create a public rather than a private profile (where access must be granted manually) may have been a rushed decision not given much thought. For others, however, the meme accounts provide a platform to reach tens of thousands every day. Although we found few advertisements in our material, the potential for economic gain adds yet another ethical dimension to the online presence of healthcare professionals. In contrast to the collapse of temporal and spatial distance, the memes commonly preserve a social distance by using medical terminology, requiring detailed medical knowledge to “get it” or by referring to situations unique to healthcare professionals. It is likely that this exclusiveness makes the memes able to strengthen the sense of group identity among healthcare professionals (7). One may also argue that this social distance mitigates the potential for harm as it makes the contents of the memes less accessible and understandable for people outside healthcare professions. The official presence of governmental bodies and healthcare institutions on the same platform – possibly serving contents side-by-side the anonymous accounts – is yet another example of unclear borders that may give the memes unwarranted legitimacy.

Overall, this study has demonstrated that patients play a peripheral role in the healthcare-associated social media memes but, unfortunately, close to 5% of the included memes were regarded as offensive. The characteristic features of these offensive memes were intentionally deceptive practices, which may have been deemed necessary at the time, mainly in the form of administering medications, as well as unflattering depictions of often vulnerable patient populations. Future studies are, however, necessary to investigate the concordance between the opinions of fourth year medical students, as in this study, actual patients, and experienced heath care professionals. The rapid development of new social media platforms where the borders between private and public are progressively dissolved and where algorithms select for increasingly shocking or eyebrow-raising contents, urges for further research to enable educational institutions to deal with these aspects of e-professionalism. The diversity revealed by the current study makes an open-minded approach necessary rather than abrupt condemnation. We hope that our findings can support nuanced reflections regarding positive and negative sides of healthcare-associated memes through empiric knowledge and guide the continuous refinement of e-professionalism in healthcare so that space can be found for the human sides of both patients and professionals.

### 4.1. Strengths and limitations

This study is, to our knowledge, the first broad and systematic characterization of social media memes produced by healthcare students and professionals. Norway is a country with a population who possess excellent digital skills and have wide access to social media (19, 44, 45), suggesting that both creators and the audience of the included memes are likely to be diverse and representative for a wider population. The combination of quantitative and qualitative methods enabled both broad and deep characterization of the memes. However, the approach involves important limitations. Although the study aimed to characterize the content of medical memes in an objective manner, the group of coders was small and homogenous (all medical students, both genders were represented) which could have influenced the results. To ensure consistency and trustworthiness of our results, the supervision and active participation of two senior researchers, both with experience from qualitative research and either clinical work or medical ethics, was necessary. Nevertheless, both the quantitative coding of posts and the thematic analysis involved subjective judgment. For example, the classification of posts as offensive or not revealed significant differences between coders. However, interrater agreement was found to be satisfactory, and the subjectivity of the general coding was further mitigated by removing codes lacking majority support. Humor is inherently subjective and individual, and shaped by factors such as culture, age, sex, and experience. It is therefore likely that medical students’ view on what is offensive or not that may differ from other groups, and it would have been interesting to include coders with other backgrounds, such as patients or experienced clinicians, to get a more diverse point of view. This is also the case in the thematic analysis, where it would have been interesting to involve a more heterogenous group in the discussion of the selected memes. Finally, the focused discussion only involved a selection of the posts and may thus have missed themes that were present in the larger material.

## Data availability statement

The raw data supporting the conclusions of this article will be made available by the authors, without undue reservation.

## Ethics statement

The study was approved by the Norwegian Centre for Research Data (NSD, reference number 128255) and the included accounts were notified and received written information about the study in line with privacy regulations.

## Author contributions

BM and BS provided supervision. ST and BCS developed the codes to thematically classify posts and MR, ABJ, and AHJ validated it. MR, AHJ, ST, ABJ, and BCS conducted the coding of the posts. AHJ and MR re-coded posts marked for review. ST, BCS, MR, EU, and AHJ rated posts for offensiveness. ST, BCS, AHJ, MR, ABJ, BM, and BS participated in focused discussions to qualitatively assess selected posts. AHJ conducted statistical analyses and wrote the first draft of the manuscript. All authors contributed to the design and conceptualization of the study, contributed significantly to the submitted work, and reviewed and approved the manuscript.
