# Supplementary material for: Characteristics and patients’ portrayals of Norwegian social media memes. A mixed methods analysis
Source: Front Med (Lausanne). 2023 Mar 16;10:1069945. doi: 10.3389/fmed.2023.1069945 (PMC10060973; doi:10.3389/fmed.2023.1069945)
Supplement: Supplementary file 1 [file Image_1.PDF]

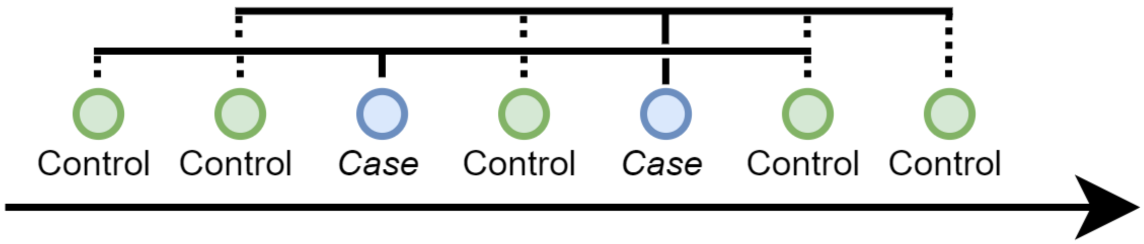

Supplementary Figure S1. Schematic illustration of how cases (patient-related posts) and controls (not patient-related posts) were selected. Each post with a specific tag (case) was compared to the two closest posts, before and after, without that tag (controls).
