# Supplementary material for: Characteristics and patients’ portrayals of Norwegian social media memes. A mixed methods analysis
Source: Front Med (Lausanne). 2023 Mar 16;10:1069945. doi: 10.3389/fmed.2023.1069945 (PMC10060973; doi:10.3389/fmed.2023.1069945)
Supplement: Supplementary file 2 [file Image_2.PDF]

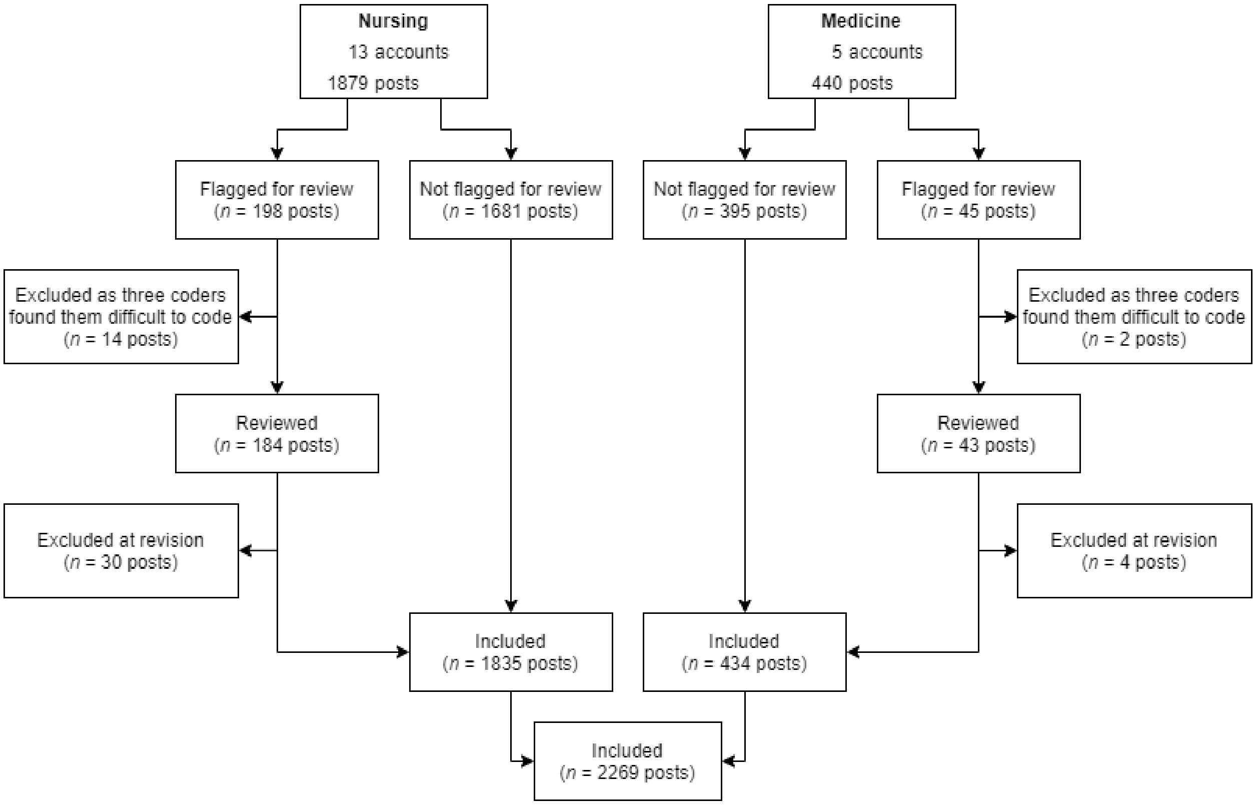

Supplementary Figure S2. Flow chart of inclusion and exclusion of posts. When uncertain, coders were able to flag posts for a second review by two authors in collaboration.
