# Supplementary material for: Characteristics and patients’ portrayals of Norwegian social media memes. A mixed methods analysis
Source: Front Med (Lausanne). 2023 Mar 16;10:1069945. doi: 10.3389/fmed.2023.1069945 (PMC10060973; doi:10.3389/fmed.2023.1069945)
Supplement: Supplementary file 3 [file Image_3.PDF]

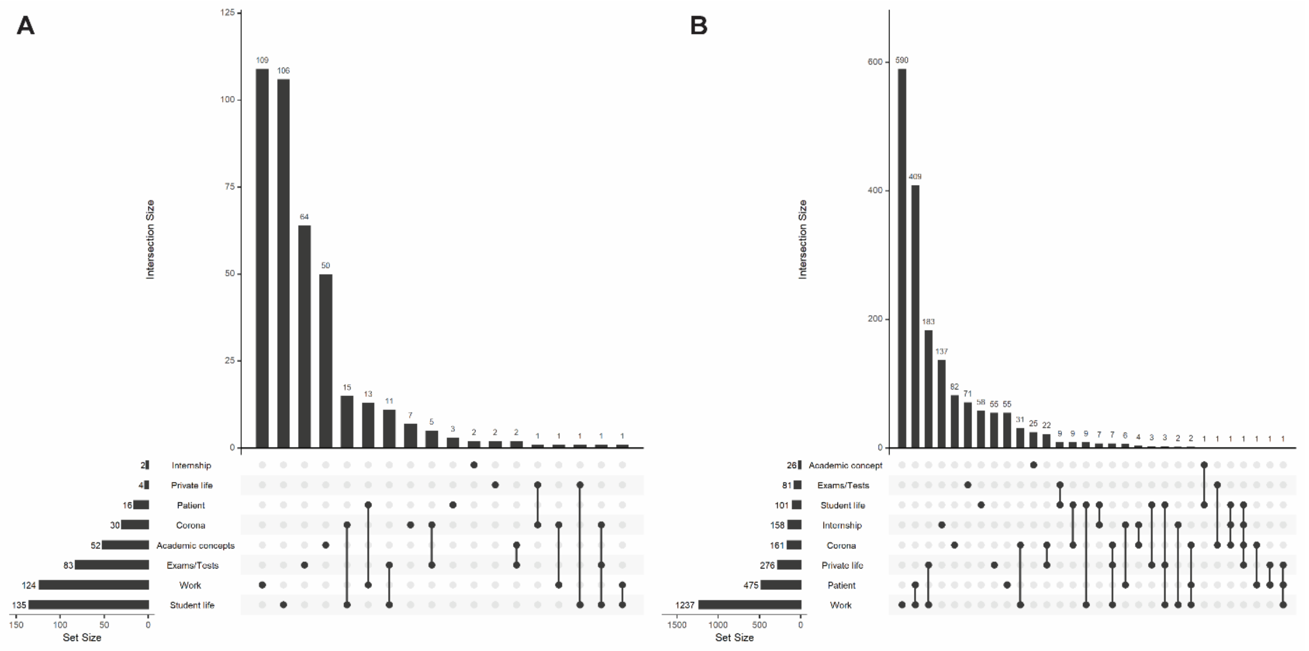


**Supplementary Figure S3.** Number of posts related to each theme, divided by (**A**) medicine-associated and (**B**) nursing-associated accounts.
