# Supplementary material for: Characteristics and patients’ portrayals of Norwegian social media memes. A mixed methods analysis
Source: Front Med (Lausanne). 2023 Mar 16;10:1069945. doi: 10.3389/fmed.2023.1069945 (PMC10060973; doi:10.3389/fmed.2023.1069945)
Supplement: Supplementary file 4 [file Image_4.PDF]

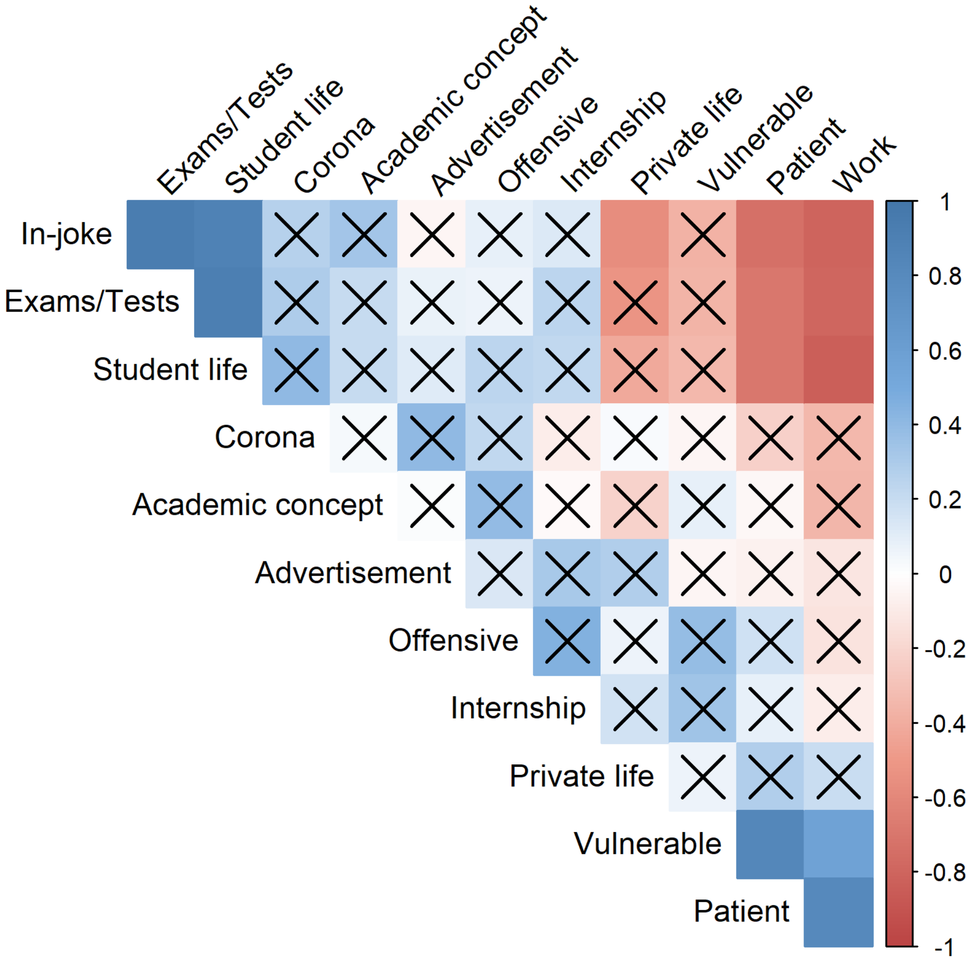

Supplementary Figure S4. Spearman correlation coefficient between themes. Non-significant correlations (adjusted $p \geq 0.05$) are crossed out.
