# Supplementary material for: Characteristics and patients’ portrayals of Norwegian social media memes. A mixed methods analysis
Source: Front Med (Lausanne). 2023 Mar 16;10:1069945. doi: 10.3389/fmed.2023.1069945 (PMC10060973; doi:10.3389/fmed.2023.1069945)
Supplement: Supplementary file 5 [file Table_1.PDF]

Supplementary Table S1. Reliability of coded themes. Disagreement refers to the number of posts where some, but not all, coders applied the corresponding theme to the post. Posts flagged for review (n = 243 posts) were assessed separately and are not included here.

**Theme**	**Gwet's Agreement Coefficient 1 (adj. *p*-value)**	**Full agreement, *n* (%)**	**Disagreement, *n* (%)**
Advertisement	1.00 (< 0.001)	2062 (99)	14 (0)
Academic concept	0.98 (< 0.001)	2008 (97)	68 (3)
In-jokes	0.98 (< 0.001)	2007 (97)	69 (3)
Internship	0.97 (< 0.001)	2003 (96)	73 (4)
Corona	0.96 (< 0.001)	1970 (95)	106 (5)
Vulnerable patient	0.96 (< 0.001)	1950 (94)	126 (6)
Offensive post	0.96 (< 0.001)	1949 (94)	127 (6)
Exams/Tests	0.95 (< 0.001)	1940 (93)	136 (7)
Private life	0.90 (< 0.001)	1823 (88)	253 (12)
Patient	0.89 (< 0.001)	1852 (89)	224 (11)
Student life	0.89 (< 0.001)	1791 (86)	285 (14)
Work	0.77 (< 0.001)	1683 (81)	393 (19)
