# Supplementary material for: Characteristics and patients’ portrayals of Norwegian social media memes. A mixed methods analysis
Source: Front Med (Lausanne). 2023 Mar 16;10:1069945. doi: 10.3389/fmed.2023.1069945 (PMC10060973; doi:10.3389/fmed.2023.1069945)
Supplement: Supplementary file 6 [file Table_2.PDF]

Supplementary Table S2. Framework for qualitative meme analysis.

Feature	Detail
Uninterpreted content	Describe who/what is present in the image/video, without any interpretation as to what it is supposed to represent.
Text	Describe any text affiliated with the image video. Also describe what the purpose of the text is; if it is a description of a situation, supposed to represent someone/something, etc.
Interpreted content	When applying interpretation, describe who/what you believe is present in the image. Describe what you think is used to represent the relevant subjects.
Humor	Describe what you think to be the joke or humor of the meme, e.g., why it is supposed to be funny. Also describe if the humor is at anyone's expense, and if so who. Are you supposed to laugh *at* someone/something, or *with* them?
Caption	Read the caption of the meme after doing the above. Does this add anything to its interpretation? If so, describe in what areas it does, and why you think it does.
Offensiveness	Describe if the meme is classified as "offensive" or "non-offensive", and why you think it is be so.
Theme	Using the above, formulate a word or short sentence describing the overarching "theme" of the meme. Try to use the same words/sentences where applicable.
